# Water-Scavenging Suspended Mediator in Electrolytes for Silicon-Based Lithium-Ion Batteries with High-Nickel Cathode

**DOI:** 10.3390/molecules31050863

**Published:** 2026-03-05

**Authors:** Siyuan Peng, Xianzheng Zhang, Weifeng Zhang, Ruiting Su, Wenwu Zou, Chenhui Pan, Limin Zhu, Li Du

**Affiliations:** Guangdong Provincial Key Laboratory of Fuel Cell Technology, School of Chemistry and Chemical Engineering, South China University of Technology, Guangzhou 510640, China; 202321023956@mail.scut.edu.cn (S.P.); 202421024057@mail.scut.edu.cn (X.Z.); 202421025234@mail.scut.edu.cn (R.S.); cezouww@mail.scut.edu.cn (W.Z.); 202321023949@mail.scut.edu.cn (C.P.); zhulimin@cecchina.com (L.Z.)

**Keywords:** H_2_O-scavenging additive, lithium-ion battery, boroxine-linked covalent organic frameworks, silicon-based anode, high-nickel cathode, suspension electrolyte

## Abstract

Trace amounts of H_2_O are inevitably introduced during lithium battery manufacturing processes, which induces the hydrolysis of LiPF_6_, leading to HF formation, which triggers a cascade of deleterious reactions that degrade the solid electrolyte interphase (SEI) and corrode electrode materials. In this work, a water-scavenging electrolyte was constructed by employing a boroxine-linked covalent organic framework (COF) as the suspended phase. The ring-opening reaction of the boroxine ring units in COFs can effectively capture H_2_O, thereby suppressing the hydrolysis of PF_6_^−^ and mitigating electrode corrosion caused by HF. Consequently, a Li-metal battery with a high-nickel cathode retained 73% of its initial capacity after 500 cycles at 1 C, and a silicon-based lithium-ion battery with a high-nickel cathode sustained stable cycling over 500 cycles at a high rate of 10 C. This suspension strategy, leveraging a boroxine-linked COF with dual H_2_O-scavenging capability, offers a scalable and versatile platform for electrolyte engineering toward practical next-generation lithium batteries.

## 1. Introduction

Lithium-ion batteries (LIBs) serve as critical power sources across a wide range of applications, from portable electronics and electric vehicles to demanding environments such as space exploration [1,2]. However, the formation of HF arising from the hydrolysis of LiPF_6_ in the presence of trace amounts of water poses a significant challenge to their electrochemical performance, a problem that is particularly pronounced in silicon-based LIBs employing high-nickel cathodes [3]. HF originating from LiPF_6_ hydrolysis attacks the high-nickel materials, causing their structure to decline, resulting in a decrease in cycling reversibility [4,5]. Silicon-based anodes are more susceptible to HF-induced corrosion than conventional graphite anodes, resulting in rapid performance decay and severe deterioration of cycling stability [6,7]. Therefore, mitigating the corrosive effects of HF by eliminating acidic impurities through rational electrolyte design represents a promising strategy toward the development of high-energy-density LIBs.

To mitigate HF formation in electrolytes, several strategic approaches have been proposed. A widely adopted approach is PF_6_^−^ stabilization: Lewis acidic species [8] (e.g., tris(pentafluorophenyl)borane) coordinate with PF_6_^−^ to suppress its dissociation into PF_5_ and LiF, thereby inhibiting HF generation. Alternatively, PF_5_ can be stabilized by nucleophilic compounds containing P, O, or N donor atoms, including trimethyl phosphite [9], diphenyldimethoxysilane [10], and dimethylacetamide [11], which effectively sequester PF_5_ through Lewis acid–base interactions and prevent its moisture-induced decomposition. A third approach focuses on the direct scavenging of HF and H_2_O, where reagents such as acid anhydrides and isocyanates chemically react with these species, removing them at the source and maintaining electrolyte “cleanliness” [12,13]. Most reported H_2_O-scavenging additives are small molecules that have been extensively explored in homogeneous electrolyte systems. In recent years, heterogeneous electrolytes have received extensive attention due to their ability to control the solvation structure. However, state-of-the-art heterogeneous electrolyte research has largely focused on mass transport and interfacial reaction phenomena in the electrical double layer (EDL). Representative strategies include leveraging the intrinsic steric hindrance of Li_2_O powders to facilitate interfacial Li^+^ desolvation [14], engineering boron nitride (BN) particles with tailored surface charge polarity to modulate ion adsorption and mass transport behavior within the EDL [15], and constructing ZnO/In_2_O_3_-derived alloyable surfaces that undergo reversible alloying reactions with metallic Li to guide homogeneous Li deposition [16]. In contrast, heterogeneous electrolyte systems remain largely unexplored for H_2_O-scavenging. The rational design of the suspended medium in the electrolyte is expected to simultaneously achieve H_2_O-scavenging and Li^+^ solvation structure regulation, representing a pivotal advance in the field. In this work, we integrate the benefits of heterogeneous systems in modulating Li^+^ solvation with targeted functional design of H_2_O-scavenging, and systematically investigate the effectiveness of this strategy in silicon-based LIBs employing high-nickel cathodes.

**Figure 1:**
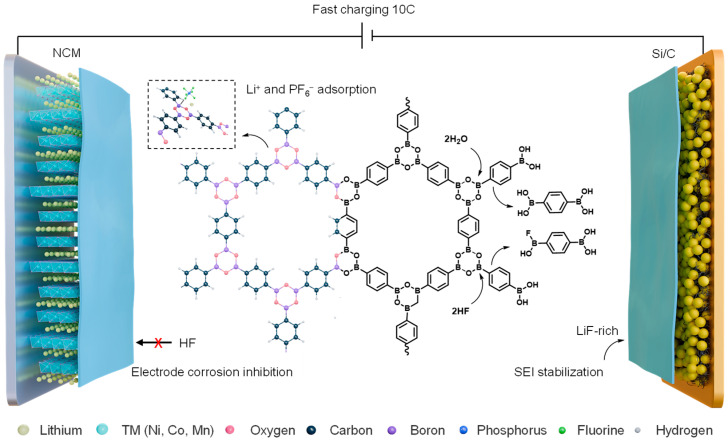
Suspended electrolyte incorporating COF-1 enables efficient H_2_O-scavenging and regulates Li^+^/PF_6_^−^ coordination, thereby suppressing HF-induced degradation and supporting stable fast-charging operations in silicon-based LIBs with high-nickel cathodes.

This study focuses on the efficacy of a boroxine-linked covalent organic framework (COF-1) in scavenging H_2_O from the electrolyte. The ring-opening reactivity of boroxine units serves as the fundamental mechanism for H_2_O-scavenging as illustrated in Figure 1. The Lewis acid–base sites within the B-O rings of COF-1 not only effectively capture H_2_O, but also enable the boron centers, acting as Lewis acid sites, to selectively adsorb and stabilize PF_6_^−^. Furthermore, COF-1 acts as a solvation structure regulator and actively participates in the construction of the electrode/electrolyte interface. As a result, both the interfacial stability and the charge transfer kinetics are significantly improved through regulating the coordination of Li^+^ and PF_6_^−^. Ultimately, the electrolyte purification suppresses electrode corrosion for high-nickel cathode materials, enabling a LiNi_0.9_Co_0.05_Mn_0.05_O_2_‖Li cell to retain >73% capacity over 500 cycles at 1 C. The stabilized SEI at the anode allows Si/C‖Li cells to maintain >95% capacity retention after 200 cycles at 1 C. The unique solvation environment created by the suspended medium reduces the Li^+^ desolvation energy barrier, which facilitates stable cycling of a silicon-based LIB with a high-nickel cathode for over 500 cycles at a high rate of 10 C.

## 2. Results and Discussion

### 2.1. Water-Scavenging Principle of Suspended Medium

**Figure 2:**
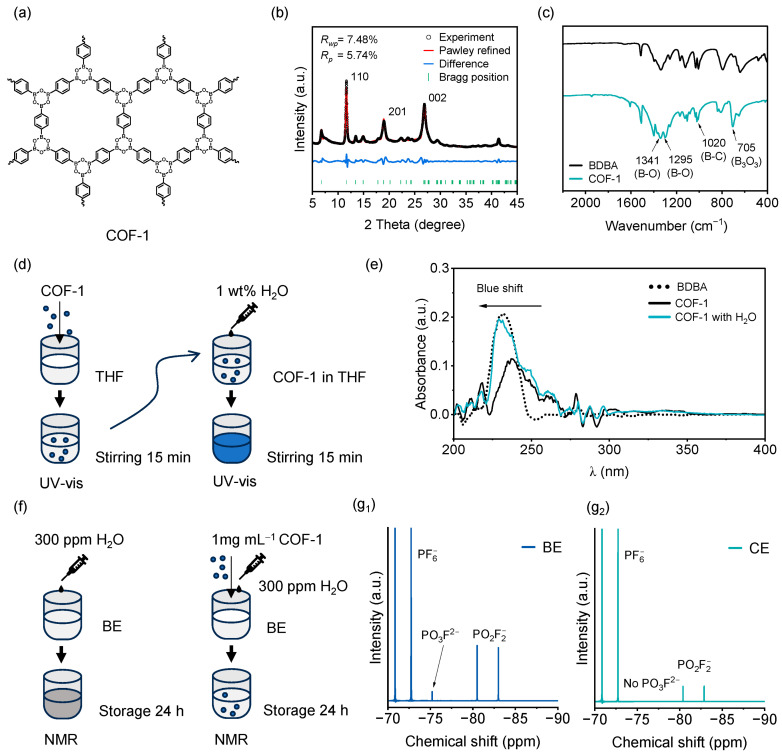
(**a**) Molecular structure of COF-1. (**b**) X-ray diffraction pattern of COF-1. (**c**) FTIR spectra of BDBA and COF-1. (**d**) Schematic of hydrolysis experiment: COF-1 was suspended in tetrahydrofuran, stirred for 15 min, and analyzed by UV-vis; then 1 wt% H_2_O was added, and suspension was re-stirred for 15 min before UV-vis measurement. (**e**) UV-vis spectra of COF-1 before and after hydrolysis. (**f**) Electrolyte hydrolysis test: 300 ppm H_2_O was added to baseline electrolyte, with one sample further containing 1 mg mL^−1^ COF-1 suspension, both stored for 24 h. ^19^F NMR spectra of (**g_1_**) BE and (**g_2_**) CE with added water.

Trace-water-scavenging additives in electrolytes can suppress the cascade of side reactions triggered by residual H_2_O. In this study, we exploit the ring-opening of the B_3_O_3_ hexacyclic units in COF-1 to simultaneously eliminate H_2_O from the electrolyte. As illustrated in Figure 2a and Figure S1, the COF-1 structure features Lewis-acidic boron sites that can accept electron pairs and Lewis-basic oxygen sites that donate lone-pair electrons. The B_3_O_3_ rings are formed by the dehydration condensation of boronic acid groups in 1,4-phenylenebisboronic acid (BDBA) under anhydrous conditions (Figure S2). In the presence of H_2_O, these rings readily undergo hydrolysis (Figure S3), reopening the cyclic structure. When all B_3_O_3_ rings are fully opened, water is consumed in a 3:1 molar ratio (H_2_O:B_3_O_3_), demonstrating the material’s efficient water-scavenging capability.

To verify successful synthesis of COF-1, the X-ray diffraction (XRD) pattern (Figure 2b) confirms its high crystallinity. Pawley refinement was performed to analyze the crystal structure, converging with low reliability factors (R_wp_ = 7.48%, R_p_ = 5.74%) and indicating excellent agreement between the observed and calculated patterns. The COF crystallizes in the hexagonal space group P6_3_/mmc, with refined unit cell parameters a = b = 15.24 Å and c = 6.65 Å (Figure S4). All diffraction peaks are clearly indexable with no impurity phases observed, confirming a well-ordered framework [17]. After being immersed in the baseline electrolyte (BE) for 120 h, the powder was collected by filtration. XRD characterization confirms that its crystal structure remained intact (Figure S5). Furthermore, the Fourier-transform infrared (FTIR) spectrum (Figure 2c) exhibits an expected absorption band at 705 cm^−1^, corresponding to the B_3_O_3_ boroxine ring, confirming complete cyclization [18]. From the SEM images (Figure S6), it can be observed that the as-prepared material exhibits a worm-like morphology with a diameter of approximately 100 nm. These results collectively demonstrate that COF-1 has been successfully synthesized, laying a solid material foundation for its further application in electrolyte.

To further elucidate the reaction mechanism between the COF and water molecules, the decomposition behavior of COF-1 in tetrahydrofuran (THF) was monitored by ultraviolet–visible (UV-vis) spectroscopy (Figure 2d). As shown in Figure 2e, COF-1 dispersed in THF exhibits a distinct absorption peak at λ = 237 nm. Upon addition of water, this peak shifts to 229 nm, indicating that the B_3_O_3_ rings undergo significant hydrolysis-induced ring-opening, which disrupts the original conjugated π-system [19]. We further evaluated the H_2_O-scavenging capability of COF-1 in a practical electrolyte (Figure 2f). ^19^F nuclear magnetic resonance (NMR) spectra (Figure 2(g_1_)) reveal that upon introducing 300 ppm H_2_O into the BE, clear characteristic peaks of PO_3_F^2−^ (δ ≈ −75 ppm) and PO_2_F_2_^−^ (δ ≈ −81 and −83 ppm) appear, which are decomposition products arising from the reaction of PF_5_ (from LiPF_6_) with H_2_O [5,20]. After introducing 300 ppm H_2_O into COF-1-containing electrolyte (CE), the PO_3_F^2−^ signal is substantially suppressed, and the intensities of the PO_2_F_2_^−^ signals are markedly reduced (Figure 2(g_2_)). The HF-scavenging effect is shown in Figure S7. Both the BE and CE with 300 ppm H_2_O show a characteristic singlet of FEC at −123 ppm and a characteristic singlet of HF at −151 ppm. The BE exhibits a significantly stronger HF signal (Figure S7a), indicating that more severe hydrolysis of PF_6_^−^ occurs in the electrolyte without timely water removal, resulting in massive HF generation. In contrast, only an extremely weak HF signal is observed in the CE (Figure S7b), verifying that the trace H_2_O and HF impurities in the electrolyte are efficiently removed by our designed COF. These results demonstrate that COF-1 effectively stabilizes PF_6_^−^ by mitigating the hydrolysis-driven decomposition pathway [3].

### 2.2. Solvation Structure Regulation Effect of COF-1

The above results clearly demonstrate the direct effectiveness of COF-1 in removing H_2_O and HF, which can be attributed to the Lewis acid–base pair characteristics of the boron–oxygen hexagonal rings. Beyond this, COF-1, as a heterogeneous medium featuring Lewis acid–base pairs, may also influence the stability of the electrode–electrolyte interface as well as the kinetics of lithium-ion transport. Investigating these effects is of critical importance for further elucidating performance enhancement under the aforementioned extreme conditions.

**Figure 3:**
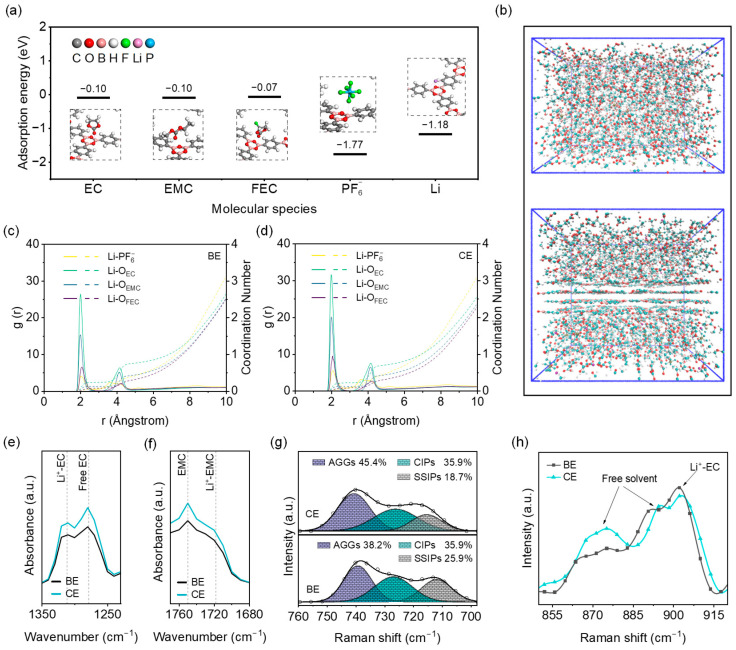
(**a**) Adsorption energies of COF-1 for EC, EMC, FEC, PF_6_^−^, and Li^+^ calculated by DFT; (**b**) Schematic of molecular dynamics simulation for solvation structure evolution; (**c**,**d**) Radial distribution functions (RDFs) for Li^+^-O (solvents) and Li^+^-F (PF_6_^−^) coordination in BE and CE, respectively; (**e**,**f**) FTIR spectra of BE and CE in regions of EC and EMC vibration modes; (**g**,**h**) Raman spectra of BE and CE showing solvent and anion coordination features.

The interactions between the COF-1 framework and electrolyte components were first investigated using density functional theory (DFT) calculations. As shown in Figure 3a, the Lewis-acidic boron sites of COF-1 exhibit adsorption toward solvent molecules (EC, EMC, FEC) as well as PF_6_^−^, with PF_6_^−^ showing the highest adsorption energy (−1.77 eV). Meanwhile, the Lewis-basic oxygen sites display a strong affinity for Li^+^, with an adsorption energy of −1.18 eV. The results indicate that boroxine rings in COF-1 selectively adsorb both cations and anions, which promotes the accumulation of PF_6_^−^ and Li^+^ at the interface and thereby facilitates the formation of contact ion pairs (CIPs). Molecular dynamics (MD) simulations were further performed to examine the evolution of the solvation structure around COF-1 (Figure 3b). The radial distribution functions (RDFs) reveal that in the presence of COF-1, the coordination of Li^+^ with solvent molecules decreases, while its coordination with PF_6_^−^ increases (Figure 3c,d and Figure S8). The results further support the hypothesis that concurrent adsorption of Li^+^ and PF_6_^−^ on the B_3_O_3_ Lewis acid–base sites promotes the formation of ion aggregates (AGGs) and CIPs (Figure S9). To experimentally verify these structural changes, FTIR spectroscopy was used to probe the Li^+^ coordination environment (Figure 3e,f). Compared with BE, CE shows an increased intensity ratio of the free EC peak at 1279 cm^−1^ (Figure 3e) and a decreased signal for Li^+^-coordinated EMC at 1718 cm^−1^ (Figure 3f), confirming reduced solvent coordination and an increased population of free solvent molecules [21,22]. Raman spectra (Figure 3g) display an enhanced peak ratio at 740 cm^−1^, corresponding to the Li^+^-PF_6_^−^ aggregates, indicating the formation of an anion-rich solvation shell [23]. Consistently, the free solvent peaks at 879 and 890 cm^−1^ intensify, while the Li^+^-EC peak at 908 cm^−1^ weakens (Figure 3h), further supporting a decrease in solvent coordination [5,24]. Finally, ionic conductivity measurements (Figure S10) show a slight decrease in CE compared to BE, which aligns with the increased formation of Li^+^-PF_6_^−^ pairs (increased AGGs and CIPs) and the corresponding reduction in Li^+^-solvents (decreased SSIPs), thereby moderating overall ion-transport kinetics [25].

### 2.3. Electrochemical Performance of Suspended Electrolyte

**Figure 4:**
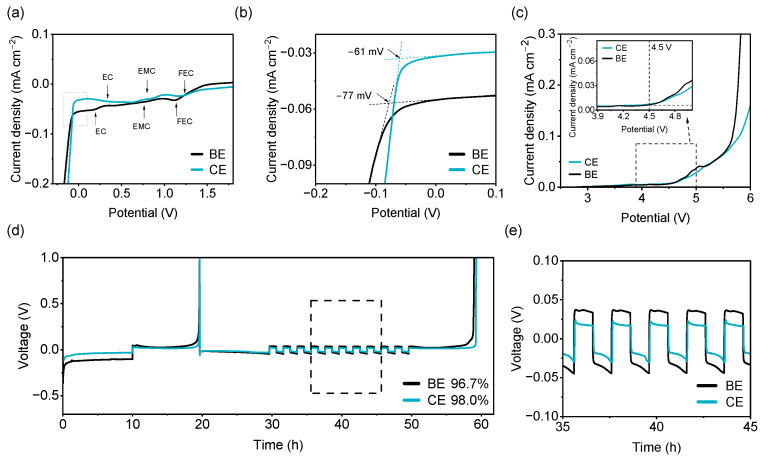
(**a**) Linear sweep voltammetry profiles of electrolyte in Cu‖Li cells at scan rate of 0.5 mV s^−1^; (**b**) Magnified view of LSV curves between −0.2 and +0.1 V, showing nucleation process of Li metal on Cu substrate; (**c**) LSV curves of electrolyte in SS‖Li cells scanned at 1 mV s^−1^, with the inset showing a locally magnified view; (**d**) Coulombic efficiency of electrolytes under Cu‖Li cells; (**e**) Overpotential of Cu‖Li cells during Li plating/stripping.

The electrochemical oxidation and reduction behaviors of CE are investigated as follows. Linear sweep voltammetry (LSV) of Cu‖Li cells from 2 V to −0.2 V (Figure 4a) shows sequential reduction peaks corresponding to FEC, EMC, and EC decomposition in BE [26,27,28,29]. In contrast, CE exhibits a lower reduction current of the solvent, which indicates that the decomposition of the solvent has been alleviated to a certain extent. When potential was swept below 0 V, lithium deposition onto the Cu substrate occurred (Figure 4b). The nucleation overpotential for Li plating is −61 mV in CE, notably lower than the −77 mV recorded in BE. This indicates that the unique solvation structure and COF-1-modified interface collectively lower the Li^+^ nucleation barrier. LSV curves of stainless steel‖Li (SS‖Li) batteries confirm that both BE and CE exhibit anodic stability beyond 4.5 V (Figure 4c). Therefore, introducing COF-1 does not compromise the inherent oxidative stability of the baseline electrolyte.

The results of coulombic efficiency measurement [30] of electrolytes are shown in Figure 4d. COF-1-containing electrolyte achieves a coulombic efficiency of 98.0%, surpassing the 96.7% of BE. Moreover, COF-1-containing electrolyte displays reduced voltage hysteresis during cycling (Figure 4e), implying not only improved interfacial stability but also faster charge-transfer kinetics.

**Figure 5:**
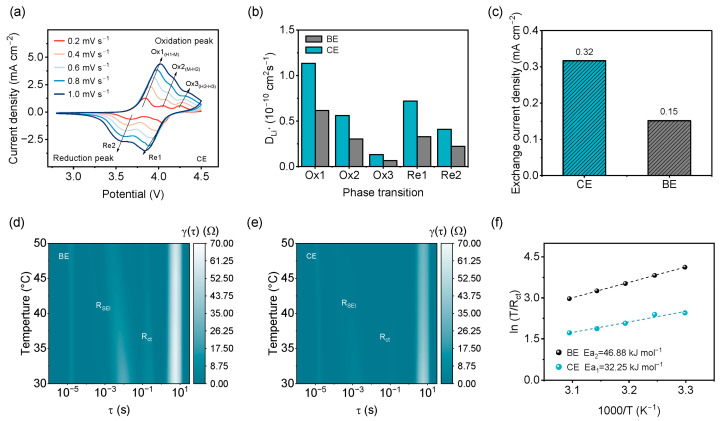
(**a**) Cyclic voltammograms of NCM‖Li cell. (**b**) Calculated Li^+^ diffusion coefficients for NCM‖Li cells. (**c**) Exchange current density (i_0_) determined from Tafel polarization plot of Li‖Li cells; Distribution of relaxation times (DRT) derived from EIS spectra of Si/C‖Si/C symmetric cells with (**d**) BE and (**e**) CE. (**f**) Fitted activation energy for charge-transfer process.

To further elucidate the kinetic advantages observed in Figure 4e, additional analyses for Li^+^ diffusion and charge-transfer processes were performed as follows. The Li^+^ diffusion kinetics in NCM‖Li cells were probed by cyclic voltammetry at various scan rates (Figure 5a and Figure S11a). The peak currents (I_p_) of characteristic redox peaks (oxidation: Ox1, Ox2, Ox3; reduction: Re1, Re2) were extracted and plotted against the square root of the scan rate (v^1/2^) in Figure S11b. Linear correlations between I_p_ and v^1/2^ are observed for all peaks, indicating a diffusion-controlled process [31]. Based on the Randles-Ševčík equation [32], the Li^+^ diffusion coefficients of NCM‖Li were derived in Figure 5b. The CE-based cell displays faster Li^+^ diffusion than the BE-based cell, and the fastest phase transition corresponds to H1-M (Ox1) [33,34]. Tafel polarization measurements on Li‖Li batteries were used to compare interfacial charge-transfer kinetics (Figure 5c and Figure S12). The exchange current density (i_0_), obtained from linear fitting of the Tafel region, is significantly higher for CE (0.32 mA cm^−2^ > 0.15 mA cm^−2^), confirming enhanced charge-transfer rates of CE. Electrochemical impedance spectroscopy (EIS) combined with distribution of relaxation times [35,36] (DRT) analysis was employed to monitor interfacial characteristics in Si/C‖Si/C symmetric cells under different temperatures (Figure 5d,e, Figures S13 and S14). Using the Arrhenius relation [26], activation energy (E_a_) for the charge-transfer process was extracted from temperature-dependent charge-transfer resistance (Figure 5f). The results demonstrate that CE substantially lowers both the charge-transfer activation energy (46.88 to 32.25 kJ mol^−1^) and the interfacial impedance at the Si/C electrode, affirming superior interfacial charge-transfer kinetics across a wide temperature range.

### 2.4. Cycling Performance of Lithium Batteries

During the battery assembly process, the moisture in the ambient atmosphere must be strictly controlled. Therefore, it is necessary to focus on the effect of COF-1-containing electrolyte under anhydrous conditions. The long-term cycling stability and rate capability of three representative cell configurations were systematically evaluated, including high-nickel Li-metal batteries (NCM‖Li), silicon-based Li-metal batteries (Si/C‖Li), and silicon-based Li-ion batteries with a high-nickel cathode (NCM‖Si/C). This work aims to elucidate the electrode–electrolyte compatibility and the regulatory role of COF-1 in these cells.

**Figure 6:**
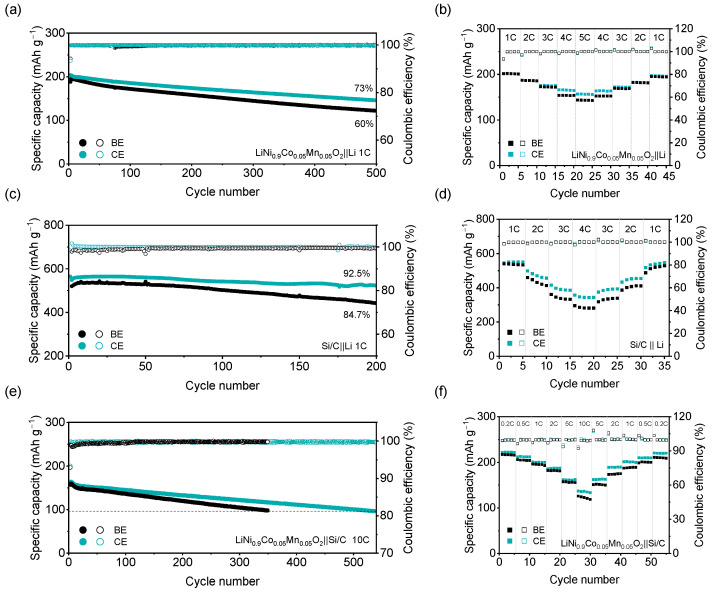
(**a**) Long-term cycling performance of NCM‖Li cells; (**b**) Rate performance of NCM‖Li cells; (**c**) Cycling stability of Si/C‖Li cells; (**d**) Rate performance of Si/C‖Li cells; (**e**) Cycle life of NCM‖Si/C full cells; (**f**) Rate performance of NCM‖Si/C full cells.

For the NCM‖Li cell (Figure 6a), long-term cycling was performed using a 1 C constant-current (CC) charge to 4.3 V followed by a constant-voltage (CV) hold and a 1 C discharge. The CE-based cell delivers an initial capacity of 204 mAh·g^−1^, and exhibits a capacity retention of 73% after 500 cycles, which is 13% higher than the capacity retention of the BE-based cell. At a high charge/discharge rate of 5 C (Figure 6b), the CE-based cell retains a discharge capacity of 156.3 mAh g^−1^, which is 9.1% higher than the 143.2 mAh g^−1^ delivered by the BE-based cell. In contrast, CE promotes an anion-enriched solvation structure and an inorganic-rich passivation layer containing abundant LiF species, which improves interfacial stability [5,37] (Figure S15). Therefore, the eventual capacity decay is attributed to gradual structural degradation of the cathode. XRD analysis further reveals that the cycled NCM cathode in CE retains a stronger (003) peak relative to the (104) peak (Figure S16), indicating that CE suppressed Li/Ni mixing [38] and improved capacity retention. Similarly, for the Si/C‖Li cell (Figure 6c), the CE achieves a capacity retention of 92.5% after 200 cycles, 7.8% higher than the BE-based cell. The stable LiF-rich interface formed in CE (Figure S17) mitigates the continuous SEI fracture/repair cycle typical of Si/C anodes [5,37], thereby reducing electrolyte depletion and improving long-term cycling stability. Given the faster charge-transfer kinetics of CE observed in Figure 5f, its rate performance (Figure 6d) is correspondingly superior: the CE achieves 550.6 mAh·g^−1^ at 1 C, retains 62.5% at 4 C (343.9 mAh·g^−1^), and recovers to 546.7 mAh·g^−1^ when the rate is restored to 1 C. In the NCM‖Si/C full cell, CE enables stable cycling for over 500 cycles at an ultrahigh charging rate of 10 C, with a capacity retention of 61.9%. By contrast, the capacity of the BE-based cell faded to 61% of the initial capacity in as few as 350 cycles. (Figure 6e). Even under stringent 10 C charge/10 C discharge conditions for the rate measurement in Figure 6f, the CE-based full cell maintains a specific capacity of 136.3 mAh g^−1^, highlighting exceptional kinetics and interfacial stability.

To evaluate the efficacy of COF-1 in eliminating water under battery operation conditions, 300 ppm of H_2_O was deliberately introduced into both baseline electrolyte (BE) and COF-1-containing electrolyte (CE). The batteries in this section were all tested using the above electrolyte.

**Figure 7:**
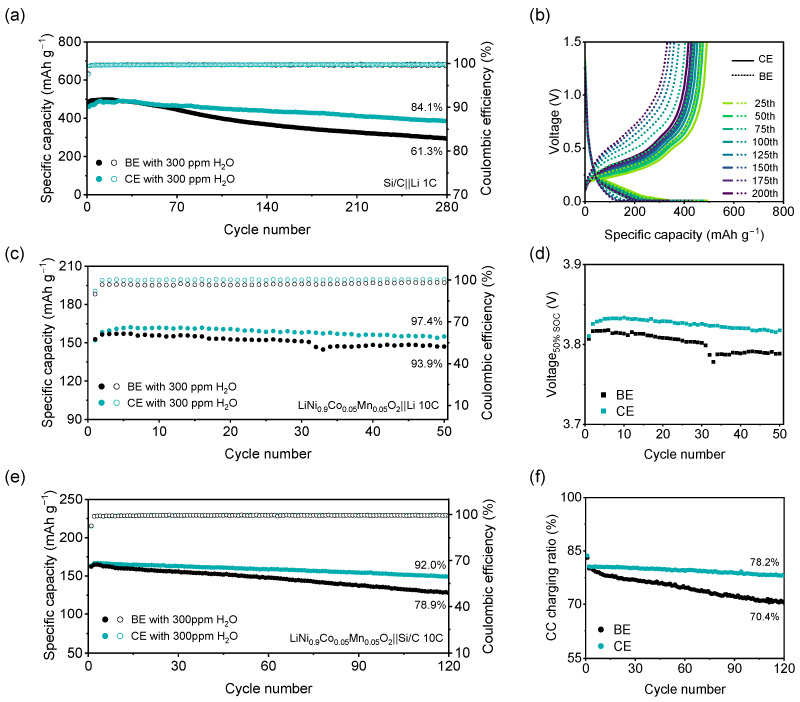
(**a**) Cycling performance of Si/C‖Li cells cycled at 1 C; (**b**) Discharge and charge curves for Si/C‖Li cells; (**c**) Cycling performance of NCM‖Li cells under 10 C charge/1 C discharge; (**d**) Discharging voltage at 50% SOC of NCM‖Li cells; (**e**) Cycling performance of NCM‖Si/C cells under 10 C charge/1 C discharge; (**f**) Charging capacity ratio of CC in NCM‖Si/C cells.

Si/C‖Li half-cells were assembled to assess the impact of H_2_O on the Si/C anode. As shown in Figure 7a, the cell employing CE delivers a capacity retention of >84.1% over 280 cycles at 1 C, whereas the cell using BE suffers rapid capacity decay, retaining only 61.3% of its initial capacity due to water-induced corrosion of the Si/C electrode. The CE-based cell also exhibits high average coulombic efficiency (99.72%), reflecting slow interfacial degradation. In addition, charge/discharge plateaus in the capacity–voltage curve exhibit a larger voltage hysteresis for BE, and the discharge capacity ratio of the constant-current (CC) reached 53.0% for CE, 6.8% higher than that of BE (Figure 7b and Figure S18). Therefore, lower polarization and enhanced charge-transfer efficiency are further confirmed for CE. To probe the benefit of water removal on a high-nickel cathode, NCM‖Li cells were assembled with the same water-containing electrolytes. Under 10 C fast-charging and 1 C discharging (Figure 7c), the CE-based cell maintains a capacity retention of 97.4% over 50 cycles, whereas the BE-based cell retains only 93.9%. The discharge voltages at 50% SOC in Figure 7d reveal that CE maintains a higher potential than BE, and effectively suppresses the polarization of the NCM‖Li cell. Finally, the performances of full cells (NCM‖Si/C) are shown in Figure 7e. The CE-based cell cycles over 120 cycles with a capacity retention of 92.0%, representing an improvement of 78.9% over the BE-based cell. Moreover, the charging capacity ratio of CC in the last cycle increases from 70.4% to 78.2% with CE (Figure 7f). These results collectively demonstrate that HF attacks both cathode and anode interfaces, and that the incorporation of COF-1 effectively scavenges H_2_O, mitigates electrode degradation, and thereby enhances the overall capacity retention of the cell.

The factors that affect battery capacity ultimately arise from the interplay of electrode structure, charge-transfer kinetics, and interfacial stability [39]. COF-1, as a suspended functional medium, not only accelerates charge-transfer kinetics but also enhances the thermodynamic stability of the interface. It thus offers a balanced performance profile—in capacity, rate, and cycling stability—across diverse battery configurations. Through its dual functions of electrolyte self-cleansing and solvation structure regulation, COF-1 provides a promising design paradigm for achieving long-cycling, high-energy Li-ion batteries.

## 3. Materials and Methods

### 3.1. Synthesis of COF-1

1,4-Phenylenebisboronic acid (BDBA) (132.6 mg, 0.8 mmol) was dissolved in a 1,4-dioxane/mesitylene (1:2, *v*/*v*) mixed solvent (8 mL) in a 10 mL Pyrex tube. The mixture was sonicated for 10 min, flash-frozen in liquid nitrogen, and subjected to three freeze-pump-thaw cycles under argon. The tube was then sealed and heated at 120 °C for 3 days. The resulting precipitate was isolated by filtration and washed with tetrahydrofuran. After vacuum drying at 120 °C overnight, COF-1 was obtained as white powder (65.5 mg, 63.2% yield).

### 3.2. Materials and Electrolytes

The baseline electrolyte (BE) consisted of 1.0 M LiPF_6_ in EC/EMC (3:7, *w*/*w*) with 10 wt% fluoroethylene carbonates (FEC), obtained from Nanjing Modges Energy Technology Co., Ltd. (Nanjing, China). The COF-1-containing electrolyte (CE) was prepared by dispersing ball-milled COF-1 into the BE at a concentration of 1.0 mg mL^−1^.

Cathode slurries were prepared by dispersing LiNi_0.9_Co_0.05_Mn_0.05_O_2_, Super P and PVDF (purchased from Shenzhen Kejing Star Technology Co., Ltd., Shenzhen, China) in NMP solvent at a mass ratio of 8:1:1. The mixture was stirred for 6 h and then uniformly cast onto carbon-coated aluminum foil. The electrodes were vacuum-dried at 80 °C for 12 h. The typical areal mass loading was 3.4 mg cm^−2^.

Anode slurries were prepared by dispersing Si/C, Super P and PAA-Li. (purchased from Shenzhen Kejing Star Technology Co., Ltd., Shenzhen, China) in H_2_O at a mass ratio of 9:0.5:0.5. The mixture was stirred for 6 h and then uniformly cast onto Cu foil. The electrodes were vacuum-dried at 60 °C for 12 h. The typical areal mass loading was 1.7 and 3.0 mg·cm^−2^.

### 3.3. Electrochemical Measurements

The electrochemical properties were characterized using an Autolab workstation (Metrohm, Herisau, Switzerland). Linear sweep voltammetry measurements were performed on SS‖Li cells at 1 mV s^−1^ (2.5–6 V). Cyclic voltammetry measurements were conducted using an IGS4030 (Ingsens Instruments, Guangzhou, China) instrument under the following conditions: (1) NCM‖Li system at 0.2–1.0 mV s^−1^ (2.8–4.5 V) and (2) Cu‖Li system at 0.5 mV s^−1^ (2.0 to −0.2 V). Ionic conductivity was evaluated using electrochemical impedance spectroscopy on SS|PE|SS cells over a frequency range from 100 kHz to 0.1 Hz. *σ* was calculated using the equation $\sigma =\frac{L}{RS}$ where *L* is the PE membrane thickness, *R* is the bulk resistance derived from the Nyquist plot, and *S* is the electrode–electrolyte contact area. The variable temperature EIS of Si/C‖Si/C cells was conducted within the temperature range of 30 °C to 50 °C, with a 5 °C interval. The test frequency range was from 10^6^ Hz to 10^−1^ Hz, with an AC amplitude of 10 mV. The galvanostatic charge/discharge cycling of cells (Si/C‖Li, NCM‖Li, NCM‖Si/C) was performed using a LAND-CT3001A battery tester (Wuhan LAND Electronic Co., Ltd., Wuhan, China) or equivalent.

### 3.4. Density Functional Theory Calculations

Density functional theory calculations were performed using the Dmol3 module in Materials Studio 2023 (BIOVIA, San Diego, CA, USA) [40] and employing PBE functional with generalized gradient approximation (GGA) [41,42,43]. Convergence criteria were set to 1 × 10^−5^ Ha for energy and 0.002 Ha/Å for forces. Van der Waals interactions were accounted for using the Grimme [44] correction method.

### 3.5. Molecular Dynamics Simulations

All molecular dynamics (MD) simulations were performed using the Gromacs package [45] (version 2022). The initial configurations and force field parameters for the simulations were based on GAFF [46] and UFF [47] force fields via Sobtop [48]. Atomic charges for small molecules were determined by fitting RESP charges in Multiwfn [49,50] based on the wavefunction calculation results from ORCA [51,52,53,54], whereas REPEAT charges derived from CP2K [55] were assigned to the COF-AB. Initially, the box dimensions were 3.0 × 5.3 × 10 nm. Both systems contained 118 EC molecules, 232 EMC molecules, 37 FEC molecules, 38 Li^+^ ions, and 38 PF_6_^−^ ions. To probe the interfacial interactions, four-layer COF-1 nanosheets were incorporated into the system. In the simulations, periodic boundary conditions were applied in all XYZ directions. The Lorentz–Berthelot mixing rule was employed, and long-range electrostatic interactions were calculated using the Particle Mesh Ewald (PME) method. A cutoff distance of 1.2 nm was set for van der Waals interactions and for the real-space part of the Coulombic interactions. The integration time step was 2 fs throughout the simulation. Energy minimization was conducted using the steepest descent algorithm. To eliminate unfavorable contacts arising from the initial configuration, the system underwent a 2 ns annealing cycle, during which the temperature was raised from 300 K to 400 K and then cooled back to 300 K. Subsequently, a 5 ns NPT simulation was performed at 300 K with 1 bar applied along the z-axis of the simulation box by V-rescale thermostat and Parrinello–Rahman barostat, ensuring that the system reached the target temperature and pressure. After pre-equilibration, a 10 ns NVT production simulation was conducted, with the final 5 ns trajectory utilized for subsequent property analysis.

## 4. Conclusions

In summary, we have developed a COF-1-containing electrolyte that operates via a dual-action mechanism for silicon-based LIBs with a high-nickel cathode. The B_3_O_3_ rings in COF-1 undergo hydrolysis, effectively scavenging the trace H_2_O of the battery. Concurrently, the Lewis-acidic boron and Lewis-basic oxygen sites within the same rings synergistically adsorb PF_6_^−^ and Li^+^, respectively. This unique co-adsorption stabilizes the PF_6_^−^, suppresses its hydrolysis pathway, and promotes an anion-enriched solvation structure. Consequently, this design simultaneously addresses two critical failure modes: it mitigates HF-induced corrosion at the high-nickel cathode, and stabilizes the silicon-carbon anode interface by forming a robust, inorganic-rich SEI. The resultant full cells exhibit significantly enhanced cycling stability and rate capability, demonstrating a viable and effective electrolyte engineering strategy for high-energy batteries.

## Data Availability

The datasets generated and/or analyzed during the current study are available from the corresponding authors upon reasonable request.

## Supplementary Materials

The following supporting information can be downloaded at: https://www.mdpi.com/article/10.3390/molecules31050863/s1. Figure S1. Electrostatic potential map of COF-1 fragment. Oxygen atoms within boroxine rings exhibit a negative potential, conferring nucleophilic character, whereas boron atoms possess positive potential, rendering them electrophilic sites; Figure S2. Synthesis of COF-1; Figure S3. Digital photographs of COF-1/THF dispersion upon gradual addition of water (0.5 mg increments, total volume 3 mg; from left to right); Figure S4. Geometric model of COF-1, illustrating atomic spatial arrangement and bonding configuration of compound; Figure S5. XRD patterns of COF-1 after immersion in electrolytes for 120 h; Figure S6. Scanning electron microscopy (SEM) images of COF-1, (a) 500 nm, (b) 200 nm; Figure S7. ^19^F NMR spectra of the electrolytes: (a) BE, (b) CE; Figure S8. (a) CN values of Li^+^ coordinated with F in PF_6_^−^, and CN values of Li^+^ coordinated with O in (b) EC, (c) EMC, and (d) FEC; Figure S9. Number density of Li^+^ and F in PF_6_^−^ from (a) BE and (b) CE; Figure S10. (a) Nyquist plots measured in SS‖SS symmetric cells with BE and CE. (b) Ionic conductivity of BE and CE; Figure S11. (a) Cyclic voltammetry profile of NCM‖Li cell with baseline electrolyte; (b) The corresponding linear fits of the peak currents of CE and BE; Figure S12. Tafel polarization curves measured on Li‖Li cells; Figure S13. Nyquist plots of Si/C‖Si/C cells in (a) BE and (b) CE; Figure S14. Typical DRT analysis of Si/C‖Si/C symmetric cell. Creation of a MATLAB2025b graphical user interface for DRT toolbox is attributed to Professor Ciucci’s group [56]; Figure S15. (a–d) XPS analysis of surface of cycled cathodes; Figure S16. XRD patterns of cathodes before and after cycling; Figure S17. (a–d) XPS analysis of cycled Si/C anodes; Figure S18. Average discharge capacity ratio of constant-current (CC) process from Si/C‖Li cells.

## Abbreviations

The following abbreviations are used in this manuscript:

SEI	Solid electrolyte interphase
COF	Covalent organic framework
BDBA	1,4-phenylenebisboronic acid
THF	Tetrahydrofuran
EC	Ethylene carbonate
EMC	Ethyl methyl carbonate
FEC	Fluoroethylene carbonate
BE	Baseline electrolyte
CE	COF-1 containing electrolyte
XRD	X-ray diffraction
FTIR	Fourier transform infrared spectroscopy
UV-vis	Ultraviolet–visible spectroscopy
NMR	Nuclear magnetic resonance
DFT	Density functional theory
MD	Molecular dynamics
RDFs	Radial distribution functions
CC	Constant-current
CV	Constant-voltage
Ip	Peak current
Ox	Oxidation
Re	Reduction
LSV	Linear sweep voltammetry
DRT	Distribution of relaxation times
