# Supplementary material for: Water-Scavenging Suspended Mediator in Electrolytes for Silicon-Based Lithium-Ion Batteries with High-Nickel Cathode
Source: Molecules. 2026 Mar 5;31(5):863. doi: 10.3390/molecules31050863 (PMC12986113; doi:10.3390/molecules31050863)
Supplement: Supplementary file 1 [file molecules-31-00863-s001.zip › molecules-4172660-supplementary.pdf]

Supplementary Information

Water-Scavenging Suspended Mediator in Electrolytes for Silicon-Based Lithium-Ion Batteries with High-Nickel Cathode

*Siyuan Peng †, Xianzheng Zhang †, Weifeng Zhang *, Ruiting Su, Wenwu Zou, Chenhui Pan, Limin Zhu, Li Du **

Guangdong Provincial Key Laboratory of Fuel Cell Technology, School of Chemistry and Chemical Engineering, South China University of Technology, Guangzhou 510640, China

* *Correspondence: weifengzhang@scut.edu.cn; duli@scut.edu.cn*

† *These authors contributed equally to this work*

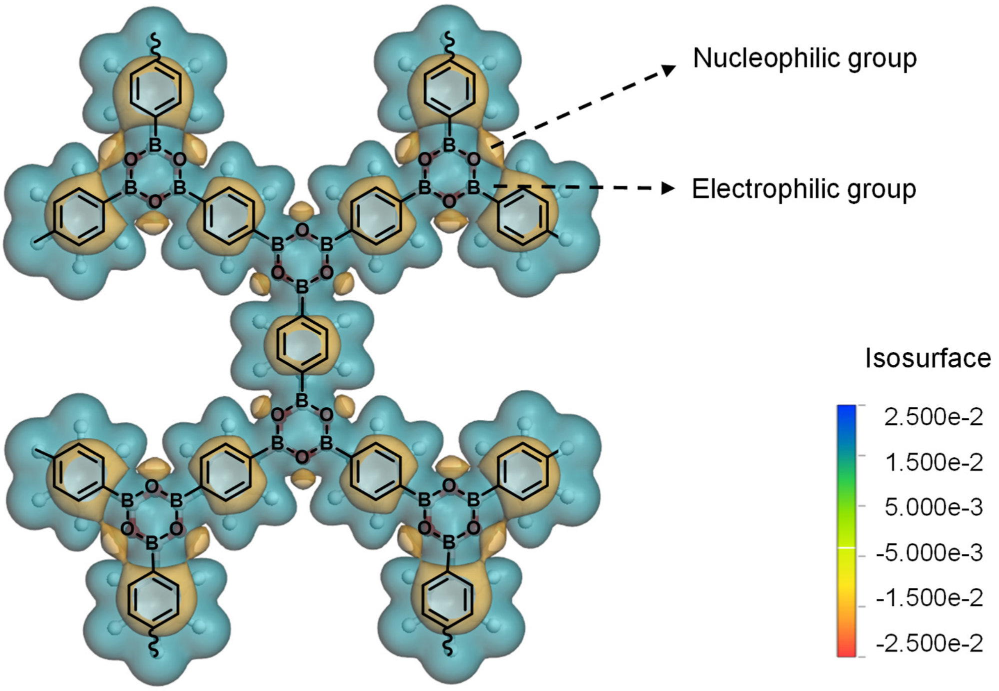

Figure S1. Electrostatic potential map of COF-1 fragment. Oxygen atoms within boroxine rings exhibit negative potential, conferring nucleophilic character, whereas boron atoms possess positive potential, rendering them electrophilic sites.

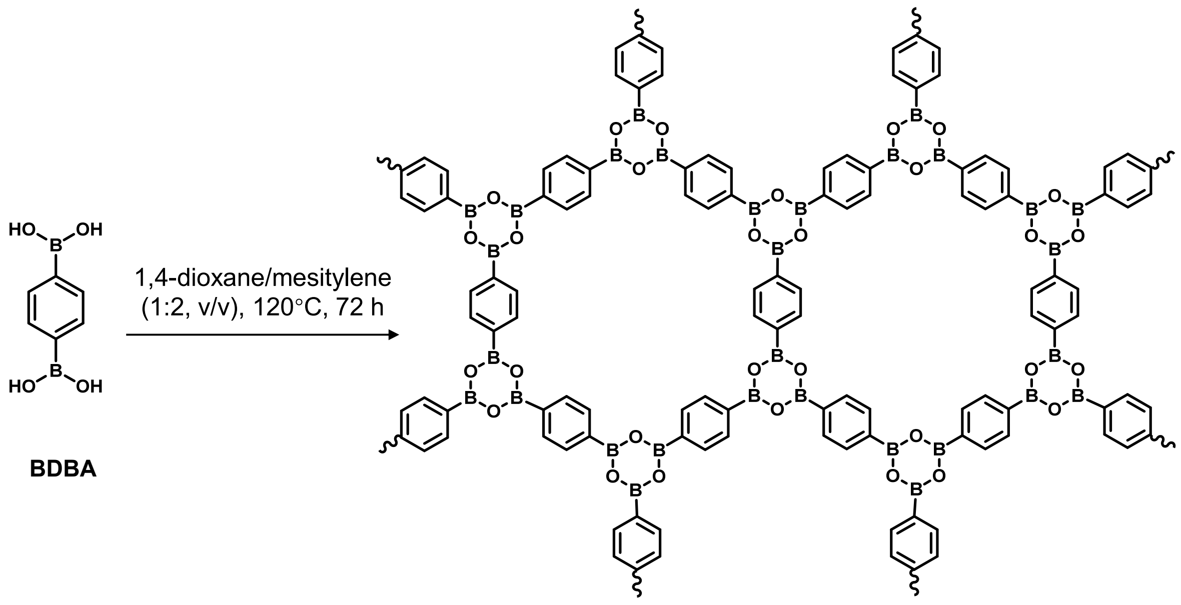

Figure S2. Synthesis of COF-1.

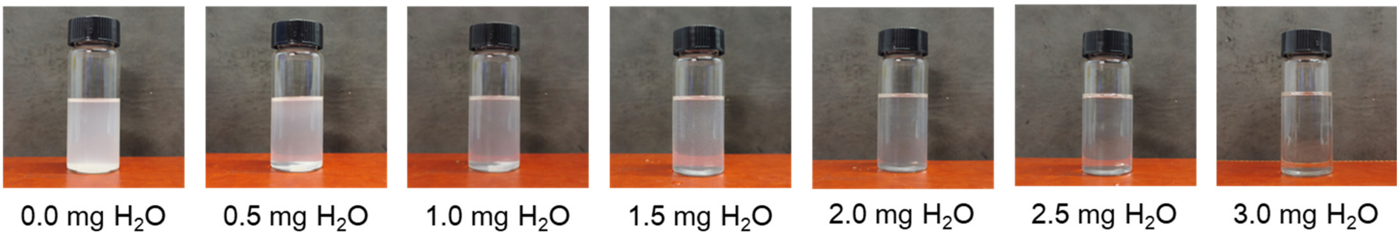

Figure S3. Digital photographs of COF-1/THF dispersion upon gradual addition of water (0.5 mg increments, total volume 3 mg; from left to right)

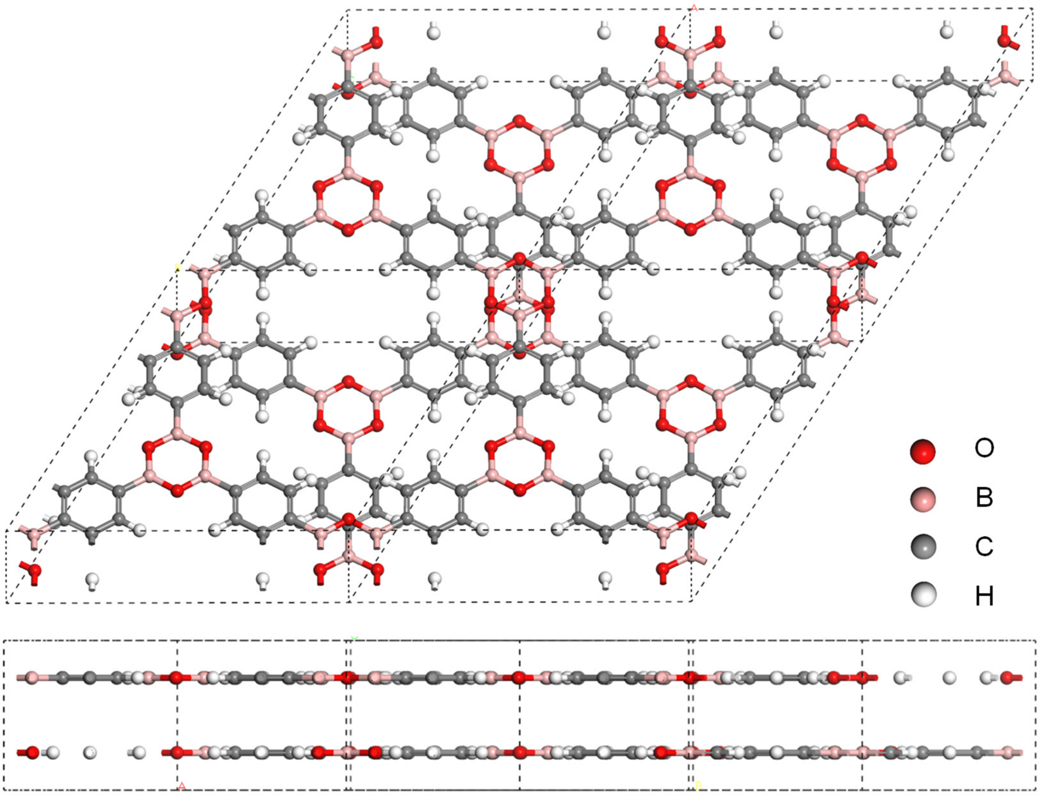

Figure S4. Geometric model of COF-1, illustrating atomic spatial arrangement and bonding configuration of compound.

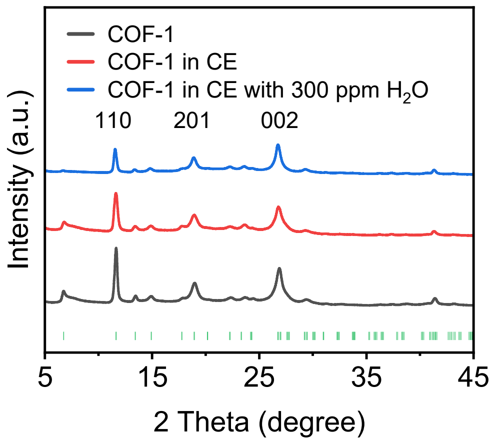

Figure S5. XRD patterns of COF-1 after immersion in electrolytes for 120 h.

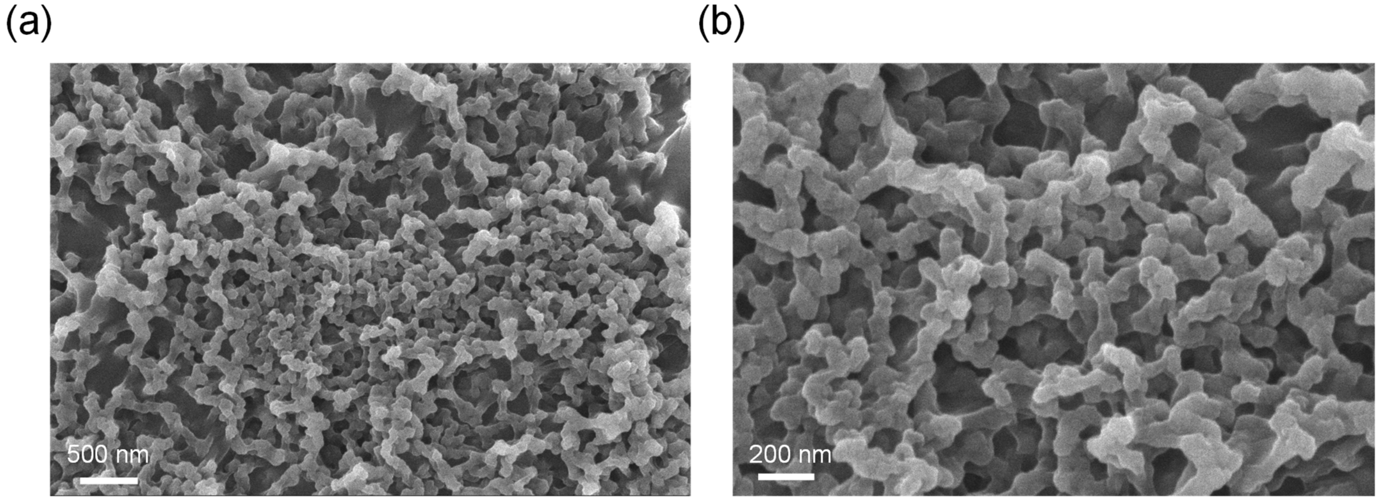

Figure S6. Scanning electron microscopy (SEM) images of COF-1, (a) 500 nm, (b) 200 nm.

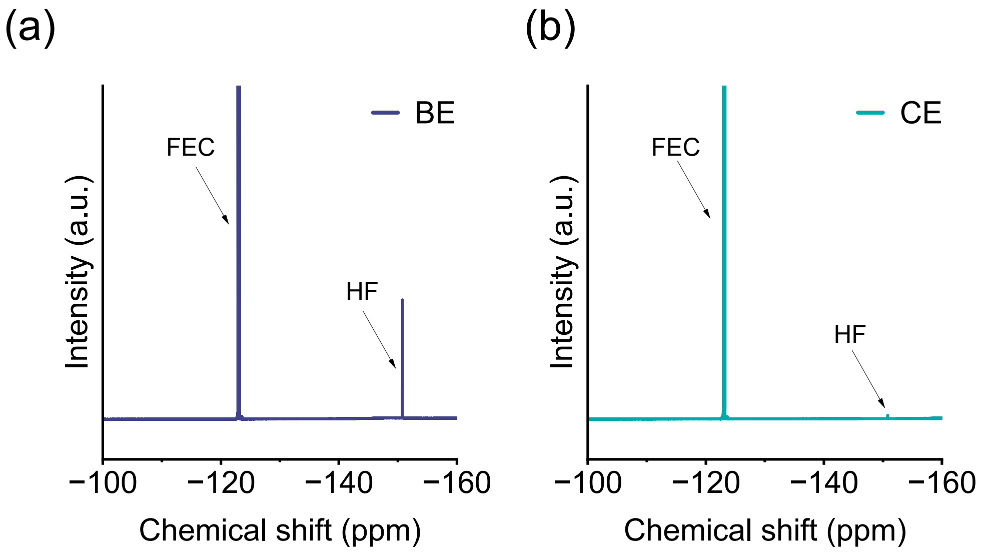

Figure S7. ^{19}F NMR spectra of the electrolytes: (a) BE, (b) CE.

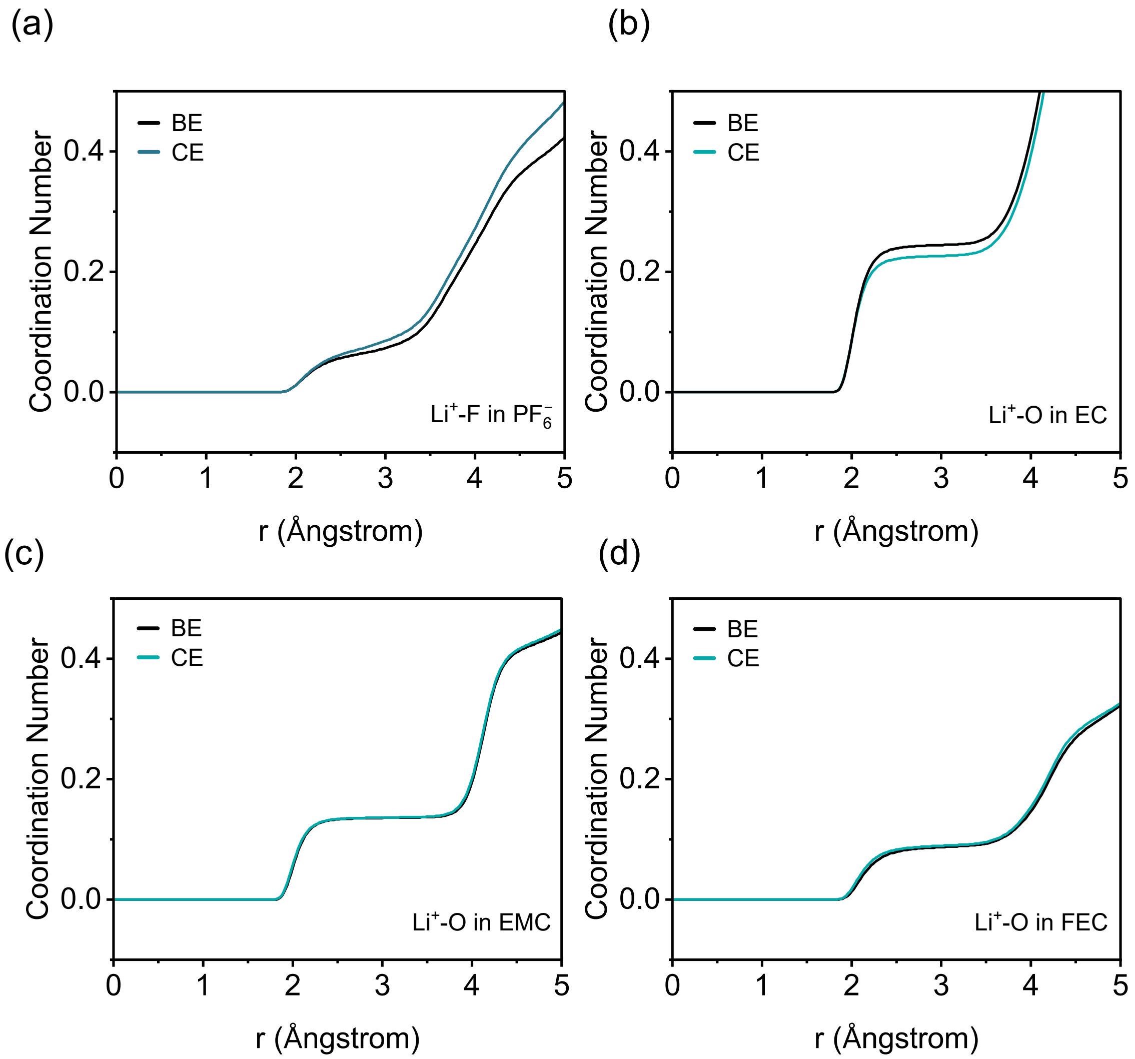

Figure S8. (a) CN values of Li^+ coordinated with F in PF_6^-, and CN values of Li^+ coordinated with O in (b) EC, (c) EMC, (d) FEC.

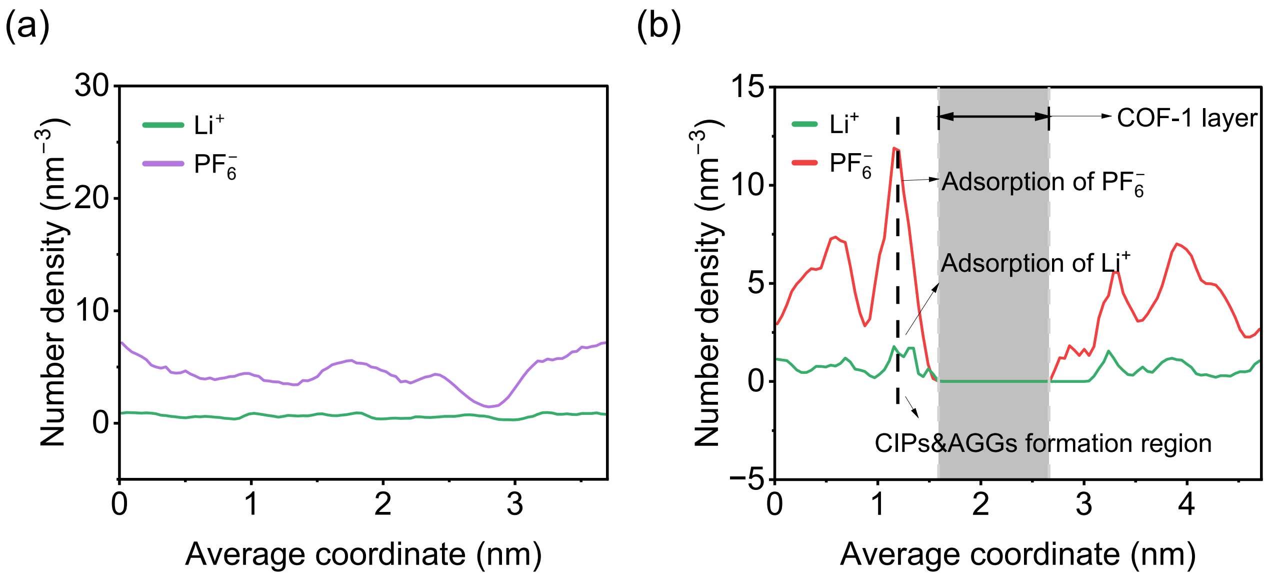

Figure S9. Number density of Li^+ and F in PF_6^- from (a) BE and (b) CE.

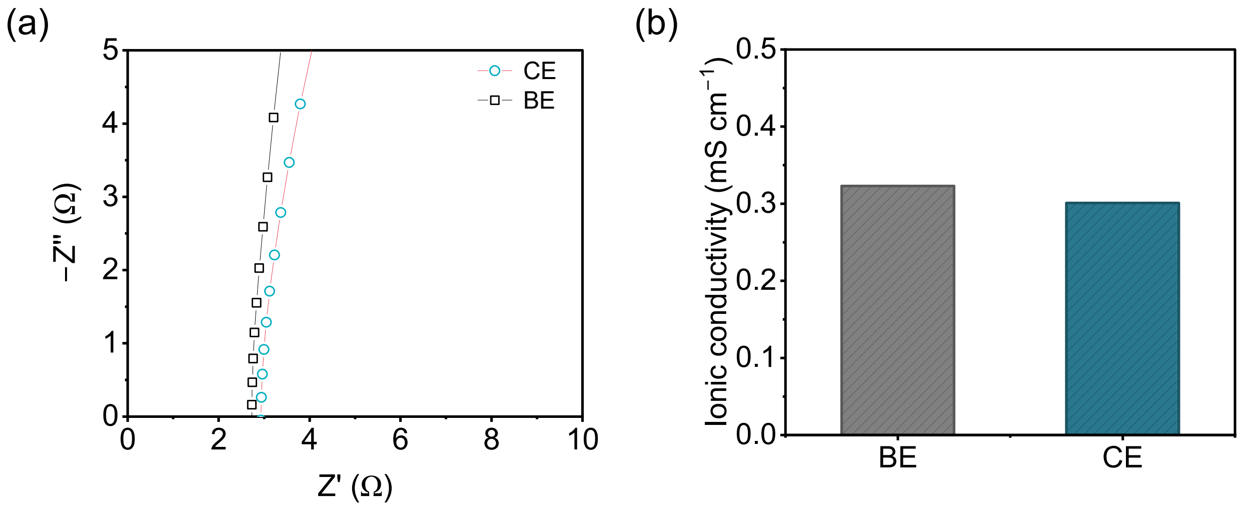

Figure S10. (a) Nyquist plots measured in SS||SS symmetric cells with BE and CE. (b) Ionic conductivity of BE and CE.

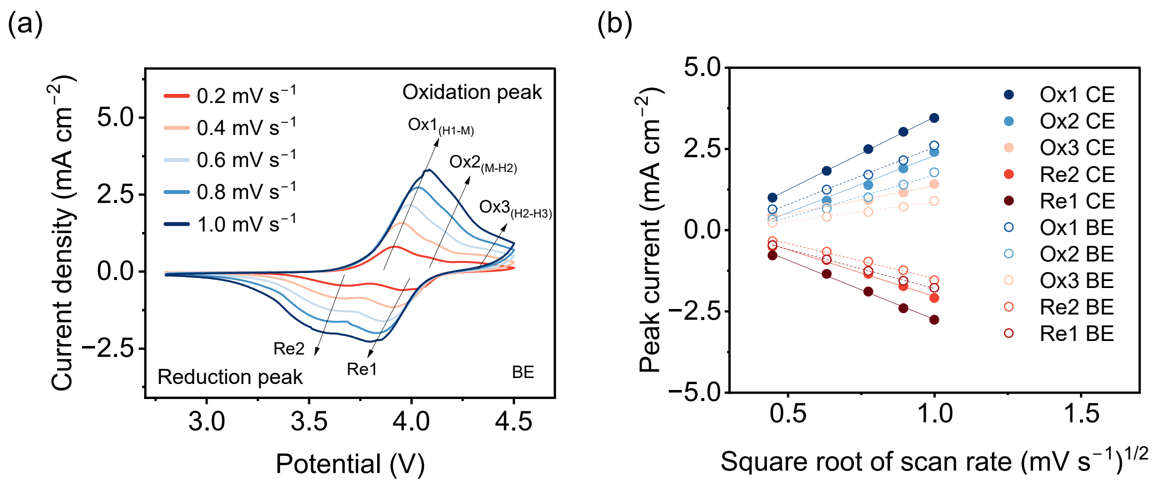

Figure S11. (a) Cyclic voltammetry (CV) profile of NCM||Li cell with baseline electrolyte; (b) Corresponding linear fits of peak currents for CE and BE.

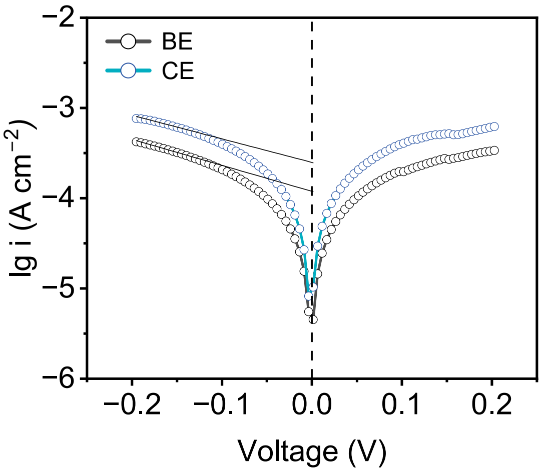

Figure S12. Tafel polarization curves measured on Li‖Li cells.

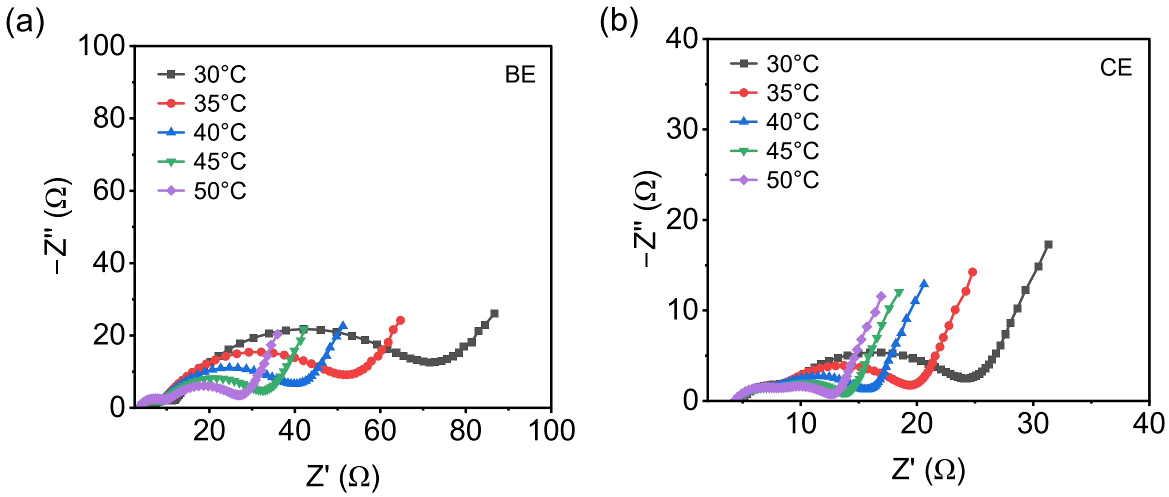

Figure S13. Nyquist plots of Si/C‖Si/C cells in (a) BE and (b) CE.

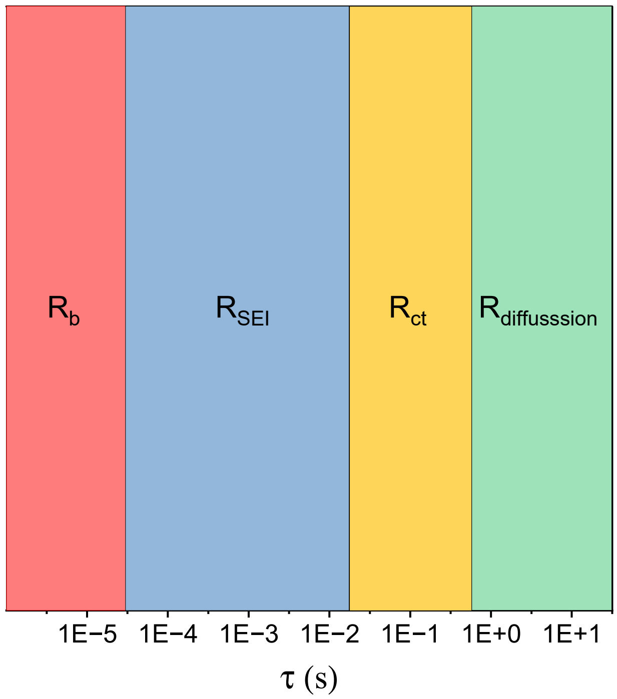

Figure S14. Typical DRT analysis of Si/C‖Si/C symmetric cell. Creation of MATLAB graphical user interface for DRT toolbox is attributed to Professor Ciucci's group [1].

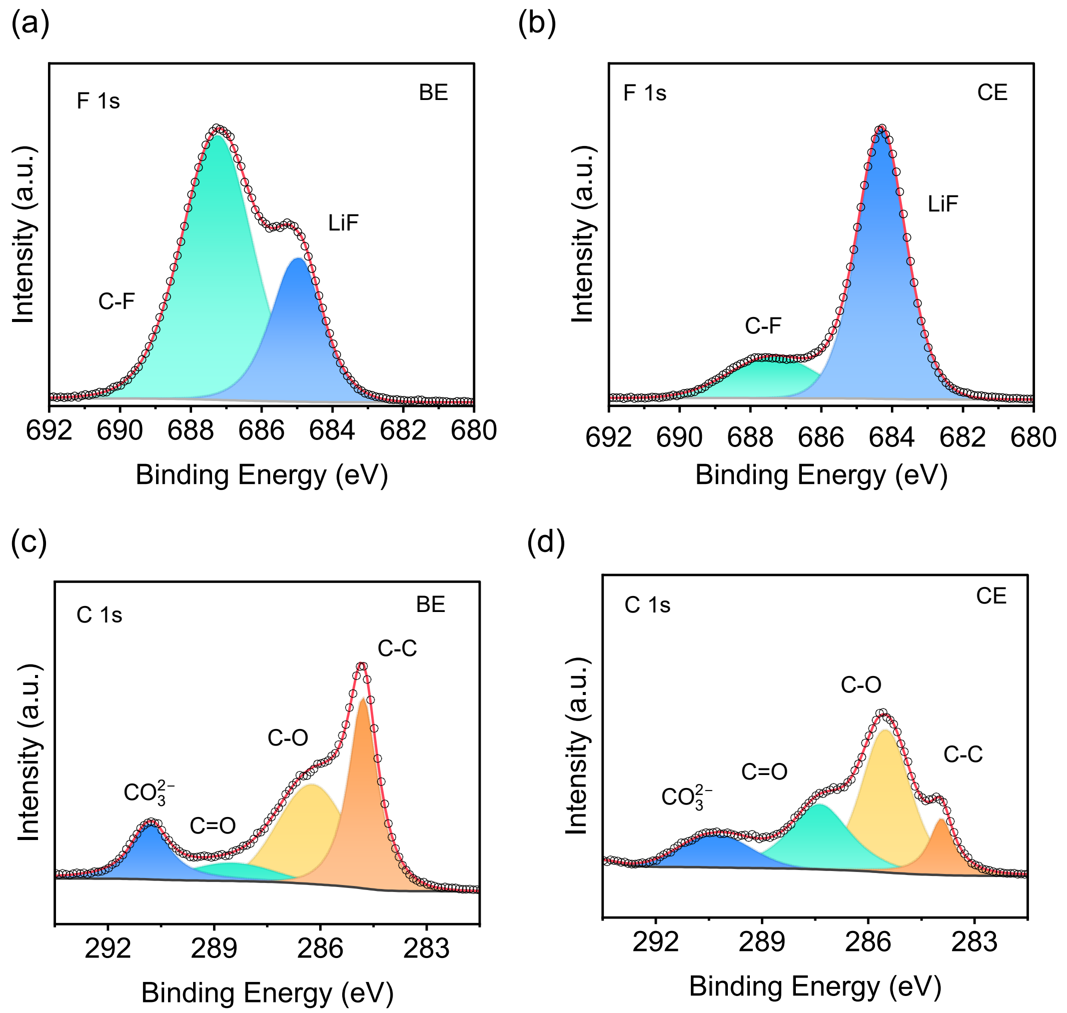

Figure S15. (a-d) XPS analysis of surface of cycled cathodes.

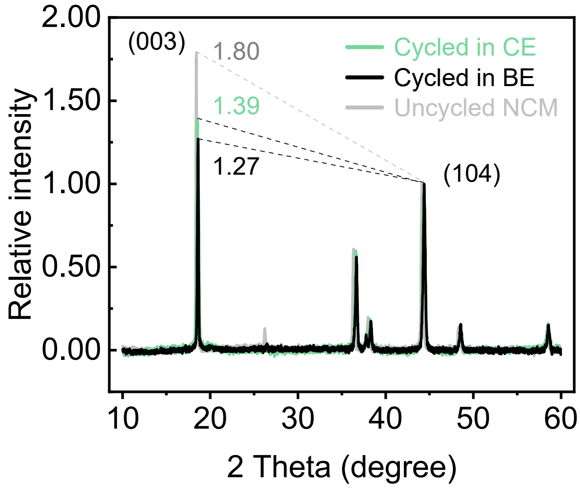

Figure S16. XRD patterns of cathodes before and after cycling.

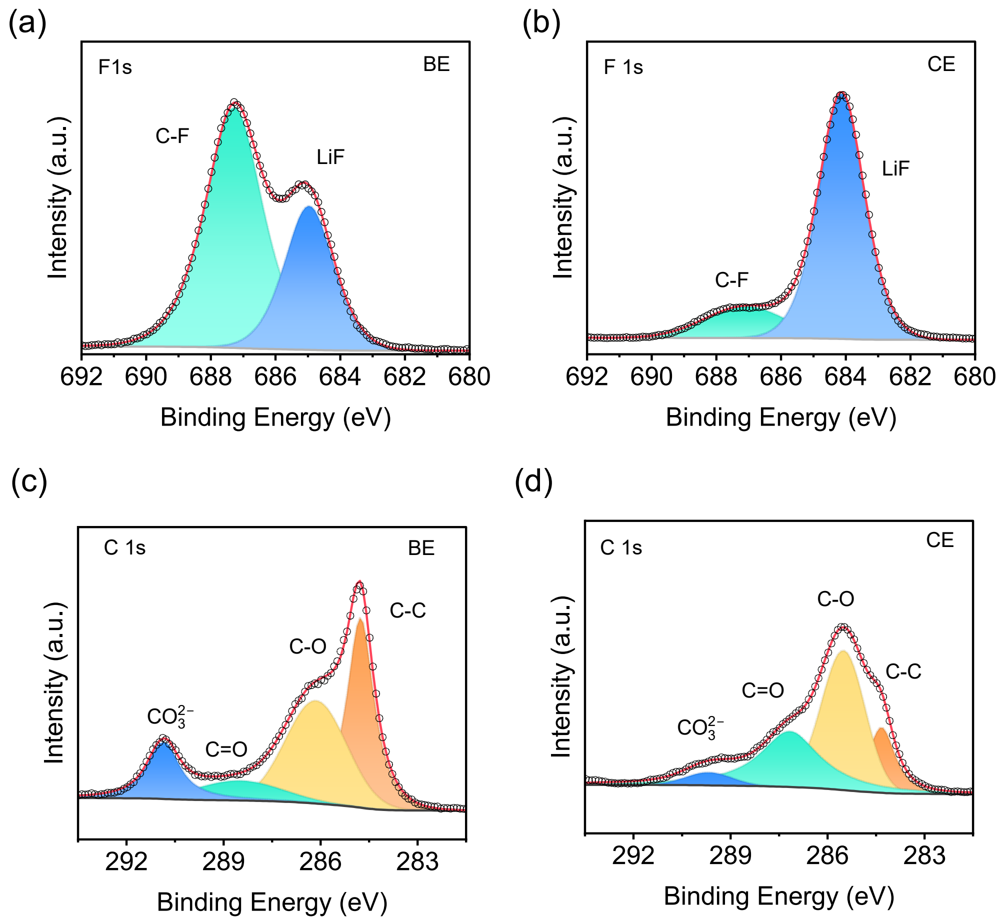

Figure S17. (a–d) XPS analysis of cycled Si/C anodes.

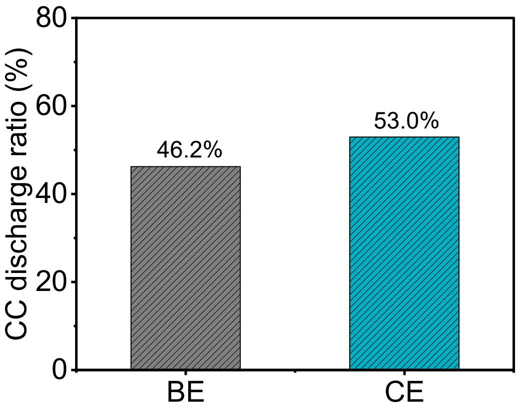

Figure S18. Average discharge capacity ratio of constant-current (CC) process from Si/C||Li cells.

Reference

1. Ciucci, F.; Chen, C. Analysis of Electrochemical Impedance Spectroscopy Data Using the Distribution of Relaxation Times: A Bayesian and Hierarchical Bayesian Approach. *Electrochimica Acta* **2015**, *167*, 439–454, doi:10.1016/j.electacta.2015.03.123.
